# Promising superabsorbent hydrogel based on carboxymethyl cellulose and polyacrylic acid: synthesis, characterization, and applications in fertilizer engineering

**DOI:** 10.1186/s13065-024-01244-w

**Published:** 2024-08-05

**Authors:** Hemat M. Dardeer, Ahmed N. Gad, Mohamed Y. Mahgoub

**Affiliations:** 1 Chemistry Department, Faculty of Science, South Valley University, Qena, 83523 Egypt; 2 Research and Development Center, Egyptian Sugar & Integrated Industries Company ‘ESIIC’, Cairo, Egypt

**Keywords:** Fertilizer, Hydrogel, Vinasse, Sugar cane, Electrical conductivity, SEM

## Abstract

The combination of hydrogel and fertilizer as slow release fertilizer hydrogel (SRFH) has become one of the most promising materials to overcome the shortcomings of conventional fertilizer by decreasing fertilizer loss rate, supplying nutrients sustainably, and lowering the frequency of irrigation. The hydrogel based on carboxymethyl cellulose (CMC) and polyacrylic acid (PAA) (CMC/PAA) was synthesized. All materials, Vinasse, hydrogel (CMC/PAA) and (Vinasse/CMC-PAA) were characterized by FTIR, XRD, and SEM. The formed hydrogel was applied to control the salinity of Vinasse to use it as a cheap and economical fertilizer. The results showed that using the prepared hydrogel with Vinasse (V/CMC-PAA) as a slow-release organic fertilizer decreased the EC value through the first six hours from 1.77 to 0.35 mmohs/cm. Also, using V/CMC-PAA can control and keep the potassium as fertilizer for 50 days. The productivity per feddan from the sugar cane crop increased by about 15%, and the number of irrigations decreased from 5 to 4 times.

## Introduction

The growing need for environmentally sustainable materials is a critical issue in the development of bio-based products at present [1–3]. The most prevalent challenges encountered in the agricultural sector include elevated soil porosity, excessive water irrigation, and inadequate fertilizer retention. Thus, the majority of conventional fertilizers are discharged into the environment via dissolution or volatilization, rendering them devoid of any advantageous effects on plants [4, 5]. The depletion of fertilizer hinders the plant's acquisition of adequate nutrients, in addition to causing environmental harm and escalating process expenses [6]. Active nutrient-containing delayed or controlled release fertilizers have been the subject of numerous studies in recent years [7]. Vinasse is a liquid byproduct that is generated during the bioethanol production process. Vinasse yields nine to fourteen liters per liter of ethanol [8]. Vinasse is a prospective bio-raw material that is utilized as fertilizer due to its nutrient composition, including nitrogen, phosphorus, and potassium, all of which have the capacity to enhance the growth of agricultural crops [9, 10]. The application of vinasse directly to the soil results in numerous environmental issues due to its elevated levels of electrical conductivity (EC), low pH, and excessive fertilization of the soil [11, 12]. Efforts are being made to supplement soil fertility with Vinasse and a byproduct of sugar cane juice processing (filter cake), which is utilized to enhance aggregate stability and average weighted diameter. Filter cake, which has a high moisture content, is an abundant source of phosphorus and organic matter [13]. However, this endeavor falls short of resolving every Vinasse issue. Vinasse must be utilized in conjunction with controlled nutrient release to the soil for the nutrients to be utilized effectively. Superabsorbent polymers (SAPs) are natural or synthetic cross-linked polyelectrolyte polymeric materials with the capacity to absorb, store, and gradually release significant quantities of water and nutrients via osmotic pressure [5, 6]. As a result, these polymers are implemented as slow release fertilizer hydrogels (SRFHs) in agricultural fields with the dual purpose of enhancing soil physical properties and reducing water irrigation rates, thereby increasing crop productivity [14, 15]. The integration of fertilizer into hydrogel results in the formation of SRFH, an organic compound that enhances soil water retention [16], reduces plant mortality, and promotes plant growth [17, 18]. Acrylic acid (PAA) or acrylate derived from synthetic SRFHs is more environmentally friendly than aldehyde and acrylamide derivatives [19, 20]. This is due to the fact that the latter undergoes numerous side reactions that produce noxious residues. Carboxymethyl cellulose (CMC) is a cellulose ether derivative [21–23] in which sodium carboxymethyl groups (–CH_2_COONa) replace a portion of the hydroxyl groups of the glucopyranose units. The food industry, pharmaceuticals, adhesives, ceramics, textiles, cosmetics, and detergents are a few of the many industries that utilize CMC [24, 25]. The carboxymethyl functional groups incorporated along the polymer chain enhanced its water solubility and reactivity. In this paper, we attempt to synthesize a new natural and synthetic polymer CMC/PAA-based combined hydrogel in order to maximize the benefits of each variety. By encapsulating vinasse with this new hydrogel, its release during irrigation can be regulated, resulting in the formation of a slow-release fertilizer that effectively reduces the detrimental effects of vinasse’s high salinity when applied as a fertilizer to sugar cane crops for the first time. CMC/PAA is implemented in order to enhance the physical characteristics, reduce the rate of release, and mitigate the adverse impacts of vinasse on the environment and soil.

## Materials and methods

### Materials

CMC-Na was prepared from bleached bagasse pulp supplied by the Qena Company of Paper Industry, Egypt. Polyacrylic acid, NaOH, sodium monoacetate, triethanolamine (TEA), and citric acid were purchased from Merck Co., Germany; methanol was supplied from Aldrich, Milwaukee, Wisconsin, USA. Vinasse is supplied from a local factory (Abo-Korkass-ESIIC) in Egypt. All chemicals were used as received without additional purification.

### Instruments

### Fourier-transformation infrared (FTIR)

Fourier transform infrared FT-IR transmission spectra of all present samples were examined in the common wavenumber range of 400 to 4000 cm^−1^ via FT-IR Nicolet 6700 spectrometer. The samples’ powder was well ground with high purity KBr and compressed into small pellets.

### X-ray diffraction (XRD)

The pulverized X-ray diffraction (PXRD) patterns of the hydrogel, Vinasse before and after coating were obtained using an advanced diffractometer instrument (Bruker D8, Germany), supported by CuKα X-ray source with a wavelength of 1.5406 Å. X-ray patterns were investigated in the 2θ range from 5° to 80° with a scanning rate 2° per min.

### Scanning electron microscope (SEM)

The surface morphology structure of Vinasse and the prepared hydrogel (CMC/PAA) were measured by scanning electron microscopy that has a type of JSM-5500-Japan apparatus, was used at magnification 10,000 times with an excitation voltage of 15 kV.

### Analytical measurements

Vinasse was characterized using proximate and ultimate analysis (LECO CHNS 932 Elemental Analyzer). Vinasse was filled into spectrophotometer cuvettes of known volume and weight. The density was detected by calculating the weight difference made after the vibration was well placed. Potassium was determined using flamephotometer, PFP 7, Jenway, UK. The Electrical conductivity was measured using conductivity meter, 4510, Jenway, UK.The efficiency of the prepared CMC/PAA as (SROFH) was followed by leaching test, where the concentration of potassium in the leached water from (SROFH-V/CMC-PAA) sample was determined compared with that from vinasse sample itself, using Flam photometer. The electrical conductivity for the two samples was measured using EC-conductivity meter.

#### Synthesis of carboxymethyl cellulose sodium salt (CMC) from bleached bagasse pulp

The procedure applied for preparing CMC is adapted as follows: first, 15 mL of sodium hydroxide (NaOH 30%) was added to 4.5 g of cellulose (bleached bagasse pulp), then 140 ml isopropanol was added to the mixture with vigorous stirring for 1 h. After that, a solution of 5.4 g sodium monochloroacetate was added with continuous stirring for 2 h at 60 °C. The solid residual was filtered off and washed with 70% three times, then dried at 60 °C. The degree of substitution of produced CMC was determined according to the ASTM D1439-03 T standard method and was found to be 0.75.

#### Synthesis of hydrogel (PAA/CMC)

Aqueous solution of CMC (1.2 wt%), containing 0.10 wt% citric acid as a crosslinker, and aqueous dispersion of PAA (0.8 wt%) were prepared in distilled water. Then, CMC solution was poured into PAA solution with stirring till the pH becomes acidic (pH ~ 5), which is neutralized with some droplets of triethanolamine TEA. The clear hydrogel was obtained. After that, the gel was placed in a petri dish and dried at 50 °C for further analysis.

#### Capsulation of vinasse on hydrogel (V/PAA/CMC)

Vinasse is the final liquid waste left over from the distillation industry of ethyl alcohol. Its importance as a fertilizer because of its highly content of potassium and organic matter. On the other hand, the using of Vinasse as fertilizer is limited due to the increased electrical conductivity (EC) of up to 25 mmoh/cm for concentrated Vinasse. At the same time, diluting Vinasse to lower the EC leads to the loss a large amount of potassium through leaching. Therefore, in this study, we are interested in controlling EC through slow fertilizer dissociation. The synthesized slow release organic fertilizer hydrogel SROFH (PAA/ CMC) uses for coating of Vinasse and improve its physical properties. The product was evaluated as a fertilizer and tracked the change in EC compared to Vinasse as a control. Generally, slow release fertilizer SRFH can be classified according to the preparation methods to two main types, matrix and coated. The matrix hydrogel can be prepared in situ method in which fertilizer present in liquid form [26], also, can be prepared with two steps in this approach the solid hydrogel is immersed fertilizer in liquid form then the swollen hydrogel is dried. The second class is coated method. In this paper we use a simple methodology for the coating method. In which Vinasse was used in solid form and the prepared hydrogel used in liquid form to be obtained good and economically SROFH (V/CMC-PAA). In this route (10 g) of the prepared hydrogel was sprayed on (100 g) of Vinasse powder with good stirring till completely wetting. After that the mixture was dried at room temperature to form capsulated particles.

### Field experiment

**Fig. 1:**
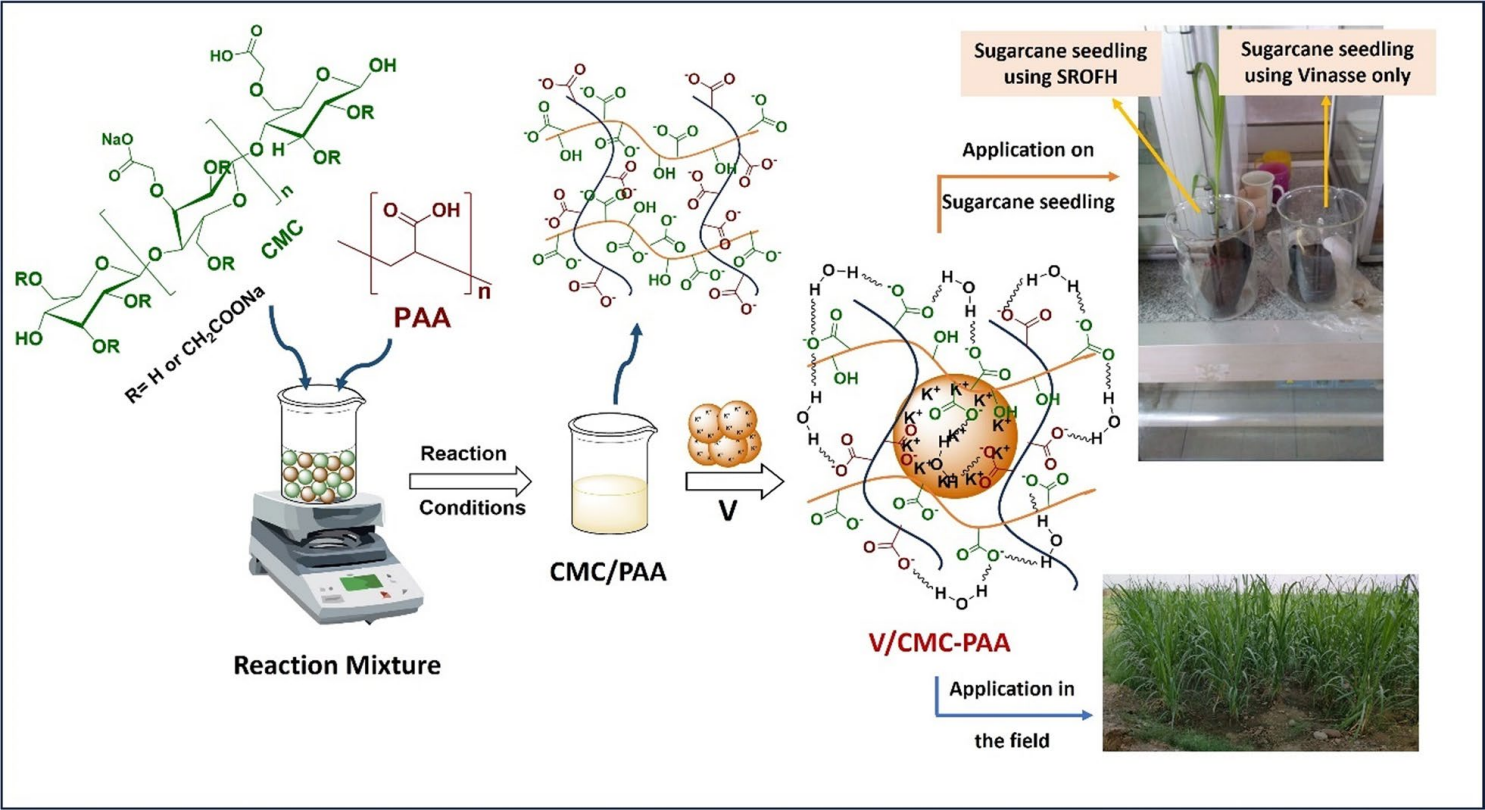
The graphic diagram for the preparation of V/CMC-PAA and its application in sugar cane crops

For cane sugar crops, the prepared V/CMC-PAA and fine dry soil were mixed in a 1:10 ratio and applied to the transplant before planting. To obtain the greatest results, the V/CMC-PAA mixture should be close to routes. Each transplant utilizes 2 g/m^2^ of a nursery bed mix of hydrogel uniformly in the top 2 inches of the nursery bed. In pot culture, mix 3–5 g/kg of soil before planting. While transplanting, mix 2 g of hydrogel per liter of water to prepare a free-flowing solution and allow it to settle for half an hour. Dip the roots of the plant in the solution and then transplant in the field [27]. In this study, we apply the experiment to two feddan plantings with cane sugar crops. Vinasse was only used in one feddan (control), and V/CMC-PAA was applied to the other feddan. In this experiment, 2 kg of hydrogel CMC/PAA have been used to coat 60 kg of Vinasse per feddan, then mixed with the soil in a 1:10 ratio. Twenty stalks of the two central raw of each crop were randomly harvested and weighed immediately. The quality control points of juice such as sugar, reducing sugar, purity and P_2_O_5_ were determined according to ICUMSA method and thus the theoretical productivity per feddan was evaluated for each treatment. All the experimental processes can be summarized in Fig. 1.

## Results and discussion

### Formation of CMC-PAA

**Scheme 1:**
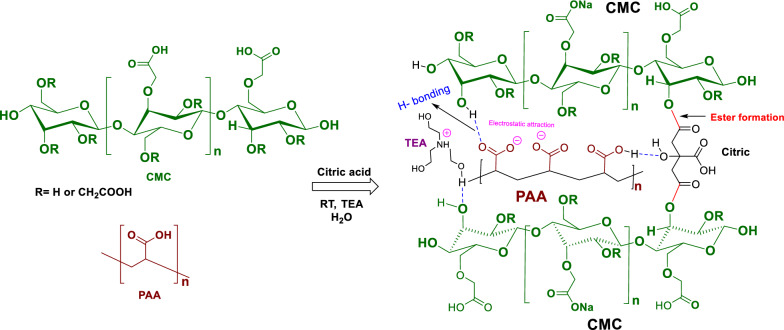
Representation of the synthetic method for the preparation of hydrogel CMC/PAA and the crosslinked hydrogel network

The chemical method for the preparation of CMC/PAA as a slow-release organic fertilizer hydrogel for vinasse involves the formation of hydrogen bonds between the active groups in CMC (COOH-OH) and the function groups in polyacrylic acid PAA (COOH). Scheme 1 indicates the synthetic method for the preparation of hydrogel and also explains the mechanism of crosslinking that takes place between citric acid (linker) and CMC and PAA [28, 29]. There are three types of crosslinking that affect the formation of the hydrogel CMC/PAA. Firstly, the ester formation between the hydroxyl groups of CMC and the carboxylic groups of citric acid. Secondly, the formation of hydrogen bonds between the hydroxyl groups of CMC, the carboxyl group of the linker (citric acid), and the carboxyl groups of polyacrylic acid [30]. The last type of crosslinking is the electrostatic attraction between negative and positive charges [31]. The formed hydrogel is very clear and has excellent homogeny. The prepared CMC/PAA is considered combined SAH because it contains natural polymer (CMC) with synthetic polymer (PAA). The CMC is grafted with PAA to improve its biodegradability and mechanical properties. The presence of CMC as a natural polymer can reduce the production costs of the hydrogel and the negative impacts on the environment. Furthermore, incorporating natural polymer with synthetic polymer lead to increase water absorbency and plant growth performance [16].

### Characterization

#### FTIR analysis

**Fig. 2:**
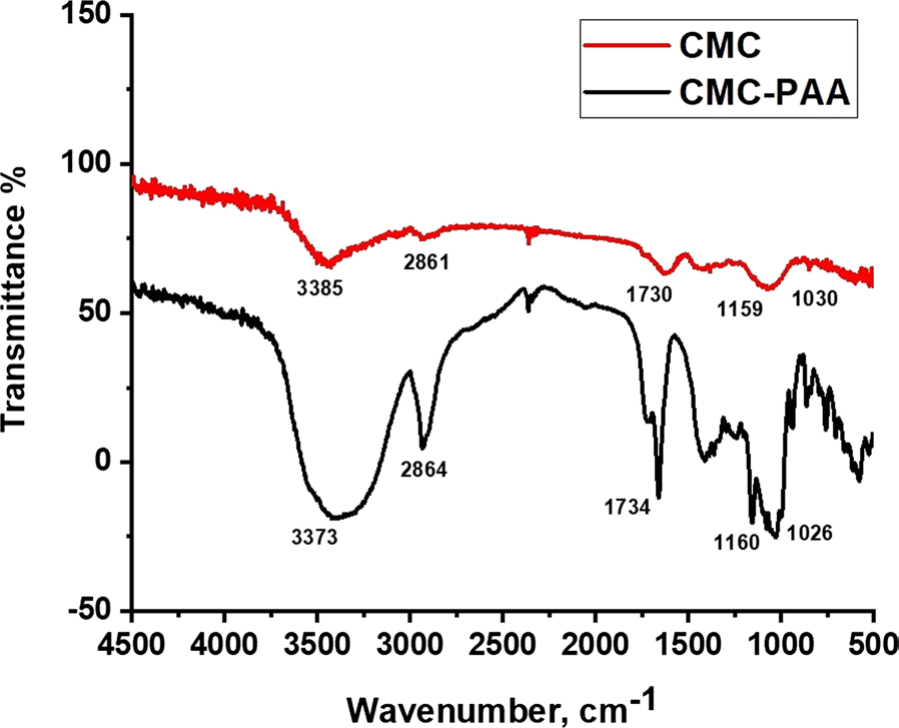
The FTIR spectra of pure CMC and CMC/PAA

**Fig. 3:**
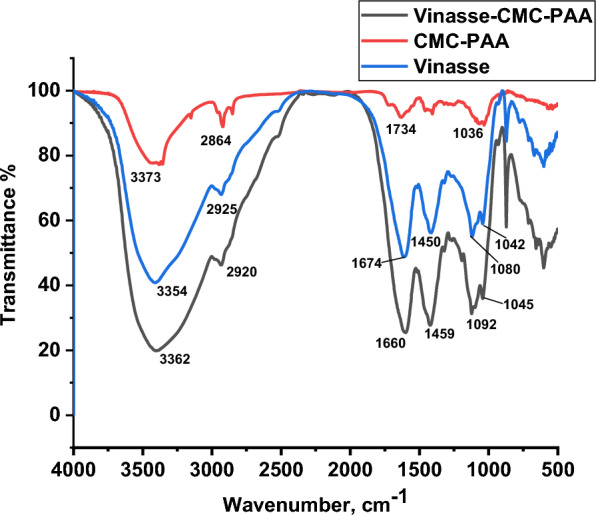
The FTIR spectra of Vinasse, CMC/PAA and V/CMC-PAA

FTIR spectra is a very important technique that is used to detect the chemical structure of target compounds (Fig. 1) displays an informative comparison between FTIR transmission spectra of pure CMC-Na and the newly prepared slow release fertilizer hydrogel (PAA/CMC). The FTIR spectrum of CMC-Na shows many distinctive peaks of carboxymethyl cellulose structure. The broadband at 3385 cm^−1^ is ascribed to stretching vibrations of OH groups as well as inter-intra molecular hydrogen bonding of CMC molecules. The characteristic band of asymmetric stretching vibration of aliphatic C–H were observed at 2861 cm^−1^ and two bands situated at 1159 and 1730 cm^−1^ were associated to the asymmetric stretching vibration of the C–O–C glycoside bridge and C=O groups. The FTIR spectrum of hydrogel (PAA/CMC) indicates some significant changes in the frequencies of the prepared hydrogel. Such these changes for the hydroxyl groups were shifted toward lower wavenumber at 3373 cm^−1^ and other characteristic transmission bands of CH aliphatic, carbonyl groups and glycosidic groups were shifted toward the higher wavenumber at 2864, 1734 and 1160 respectively, (Fig. 2, Table 1 showing the difference in the frequencies of CMC and the obtained CMC/PAA. (Fig. 3) demonstrates the comparison in the FTIR spectra of the Vinasse before and after loading on the prepared hydrogel (CMC/PAA). (Table 2) shows the changes in the intensity of PAA and CMC/PAA hydrogel. The hydroxyl groups (OH) of pure PAA were shifted toward lower wavenumber from 3390 to 3372 cm^−1^ and the peak of CH aliphatic was shifted toward lower frequency at 2864 cm^−1^, carbonyl groups were shifted from 1728 to 1734 cm^−1^ toward the higher wavenumber due to the formation of Vanderwall forces and hydrogen bonds. The chemical composition of Vinasse contains highly concentrated biodegradable organic compounds such as, residual sugar, ethanol, other metabolites resulting from parallel fermentation pathway. The FTIR spectrum of Vinasse shows a wide band at 3354 cm^−1^ indicates the absorbed water content in addition to the hydroxyl groups for residue sugar and organic acids in Vinasse [32]. Moreover, the bands at 1647 cm^−1^ are correlated to C=C and strong peak at 1450 cm^−1^ indicates C–H groups [33]. The FTIR spectrum of Vinasse after loading on the prepared hydrogel shows appearance of some significant changes in the wavenumbers according to the loading process. Such changes, the broadband at 3354 cm^−1^ in Vinasse spectrum was found to shift towards the higher wavenumber 3362 cm^−1^. Aliphatic C–H bands moved to appear at 2920 cm^−1^ towards the lower wavenumber. In addition to the C=C and C–O were shifted towards the higher wave number 1459 and 1092 cm^−1^. Table 3 indicates the difference in the intensity of Vinasse after loading on CMC-PAA hydrogel.

**Table 1:** The difference in the intensity of CMC and CMC/PAA hydrogel

Functional group	Wavenumber (cm^−1^)		Changes
	CMC	CMC/PAA	Δδ
ν[OH] symmetric	3385.42	3373.85	− 11.57
ν[CH-aliphatic]	2861.84	2864.73	+ 2.89
ν[C=O]	1730.79	1734.65	+ 3.86
ν[C–O–C]	1159.97	1160.93	+ 0.96
ν[O–H] bending vibration	1030.76	1026.55	− 4.21

**Table 2:** The difference in the intensity of PAA and CMC/PAA hydrogel

Functional group	Wavenumber (cm^−1^)		Changes
	PAA	CMC/PAA	Δδ
ν[OH] symmetric	3390.24	3373.85	− 16.39
ν[CH-aliphatic]	2890.77	2864.73	− 26.04
ν[C=O]	1728.67	1734.65	+ 5.98
ν[O–H] bending vibration	1031.72	1036.55	+ 4.83

**Table 3:** The difference in the intensity of Vinasse and Vinasse/CMC-PAA hydrogel

Functional group	Wavenumber (cm^−1^)		Changes
	Vinasse	V/CMC-PAA (HG)	Δδ
ν[OH] symmetric	3354.23	3362.43	+ 8.2
ν[CH-aliphatic]	2925.55	2920.56	− 4.99
ν[C=O]	1647.61	1660.22	+ 12.61
ν[C=C] symmetric	1450.67	1459.27	+ 8.6
ν[C–O] symmetric	1080.88	1092.34	+ 11.46
ν[C–C]	1042.45	1045.28	+ 2.83

#### XRD analysis

**Fig. 4:**
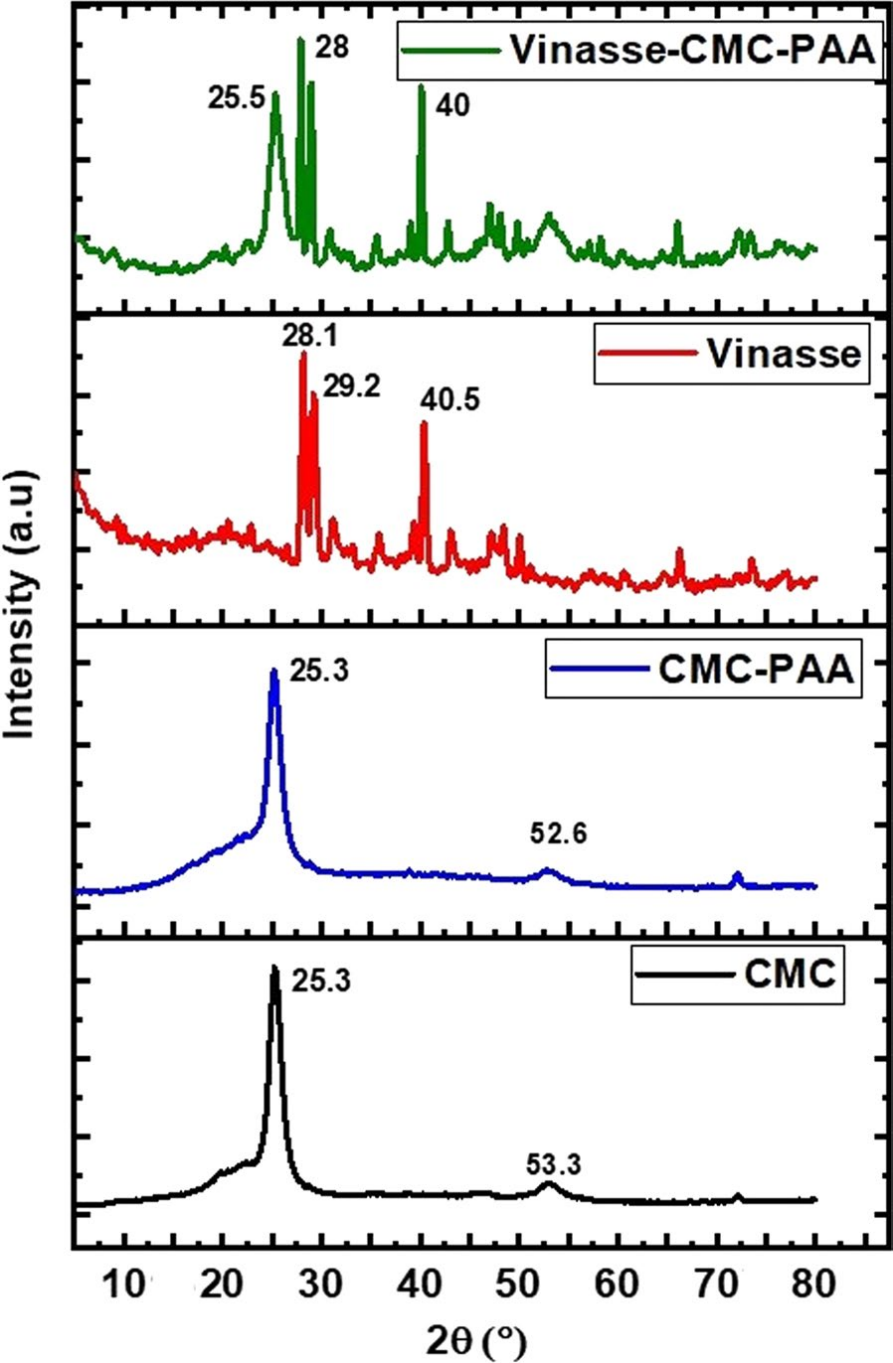
XRD patterns of all current compounds CMC, CMC/PAA, Vinasse and V/CMC-PAA

X-ray diffraction technique is a good method to examine the crystalline structure nature of compounds. In this study, XRD data was used to observe the crystallinity and amorphously value of all the prepared samples. (Fig. 4) indicates the comparison between XRD of pure CMC polymer, CMC/PAA hydrogel, Vinasse and V/CMC-PAA. The CMC spectrum illustrates two relatively sharp peaks of the semi-crystalline nature of carboxymethyl cellulose structure at 2θ = 25.3° and 53.3° [34]. The appearance of such peaks can be explained by the presence of free hydroxyl groups along the backbone of CMC and formation of hydrogen bonds between them [35]. While, the XRD pattern of the prepared hydrogel (CMC/PAA) shows that the peak at 53.3° of CMC was shifted to 52.6° and the peak became more broadening and less intensity. This is due to the formation of new hydrogen bonding between the OH, C=O groups in CMC chain and the other carboxylic groups in PAA polymer. Also, the hydrogen bonds that link the CMC chains together decreased [36]. These changes give good indication for the formation of hydrogel (CMC/PAA). The XRD spectrum of Vinasse has many sharp and intense peaks were observed at 28.1°, 29.2° and 40.5°. Furthermore, the XRD pattern of Vinasse after coating with the hydrogel (CMC/PAA) indicates presence of some changes in the intensity of the peaks. The peak at 29.2° was shifted to 2θ = 28° and the intensity of its peak became sharper. The peak at 40.5° was became 40, also, a new peak appears at 2θ = 25.5° with high intensity. These changes confirm the capsulation of Vinasse by the prepared hydrogel. This result depends on the presence of hydrophilic-hydrophilic interaction in addition to the formation of hydrogen bonding between the active groups in Vinasse and the hydroxyl groups of the hydrogel CMC/PAA. The crystalline order of these materials was V/CMC-PAA > Vinasse > CMC/PAA > CMC. These results were logical due to the fact that CMC/PAA have higher crystalline.

#### SEM analyses

**Fig. 5:**
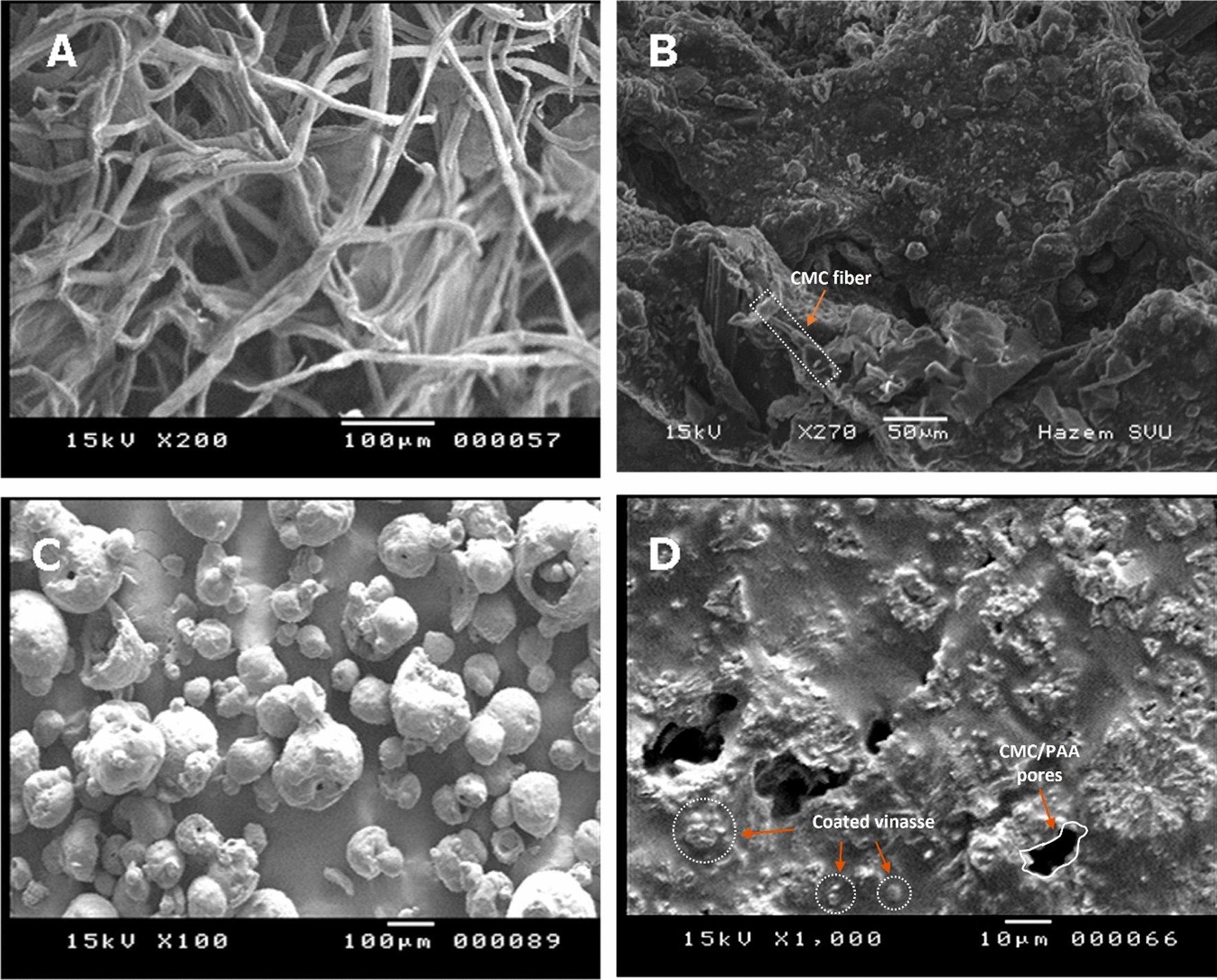
SEM images of **A** pure CMC, **B** CMC/PAA, **C** Vinasse (V), and **D** V/CMC-PAA

Figure 5 represents the indistinct morphological dissimilarities of pure CMC, CMC/PAA hydrogel, Vinasse and V/CMC-PAA samples. The surface morphology of CMC was found to be really tubes [35], as shown in (Fig. 5A). Meanwhile, (Fig. 5B) describes the surface structure of the CMC/PAA hydrogel as dark homogenous, and compact clouds with the presence of cavities and the appearance of CMC tubes. This feature gives a good indication for the synthesis of hydrogel CMC/PAA. These cavities have a vital role in the capture and storage of water. (Fig. 5C) indicates the morphological structure of Vinasse was homogenous and represented as compact white spheres in addition to small white slides and lumps. Furthermore, (Fig. 5D) illustrates the micro-topography image of the capsulation and coating of Vinasse through hydrogel. These bright white slides and spots point out the successful coating of Vinasse by hydrogel. Active absorption sites toward the soil increase in tandem with the specific surface area of the vinasse.

## Application in agriculture field

### Water absorption mechanism of CMC/PAA

**Fig. 6:**
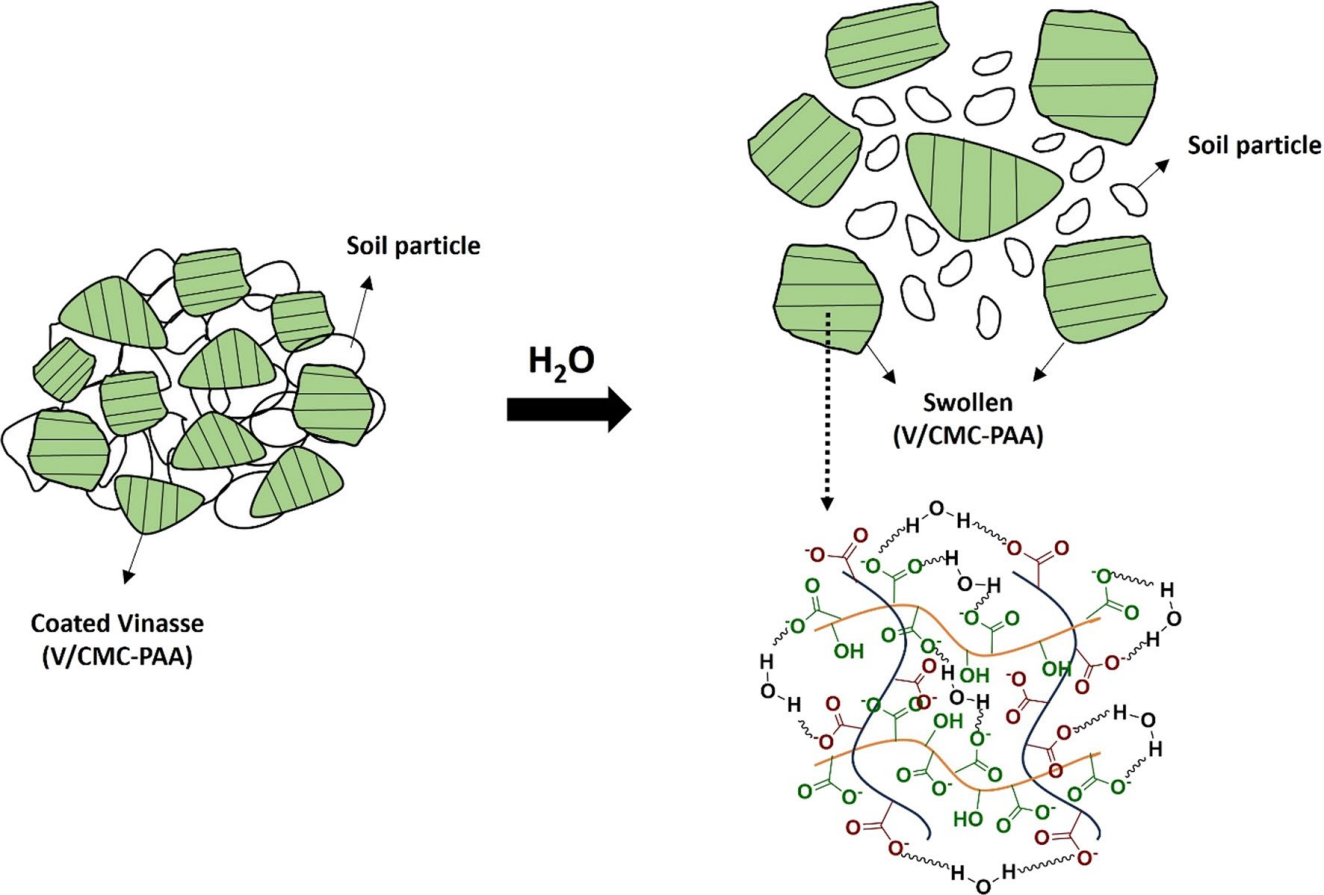
Water absorption mechanism of CMC/PAA

The main mechanism for the absorption of water by hydrogel (CMC/PAA) depend on the presence of hydrophilic groups in the hydrogel chain, such as (–COOH, –OH). The water penetrates the hydrogel system by osmosis pressure differences [13]. On the other hand, the carboxy groups that are attached to the main chain of the hydrogel (CMC/PAA) lose hydrogen atoms and leave behind negative ions that run along the main chain of the hydrogel. The repulsion that occurs between these negative charges makes the hydrogel chain attract the water molecules and bond with them by hydrogen bonding. Subsequently, it stores a large number of water molecules. This type of hydrogel can absorb more than 400 times its weight of water (Fig. 6). When it begins to dry out, the hydrogel gradually releases about 95% of its stored water and repeats this process with time [13]. It is important to note that the surface morphology plays a vital role in the adsorption mechanism of water molecules on the surface of hydrogel, as we mentioned and discussed in the SEM section (Fig. 5B). The surface sample contains caves, pores, and some cracks that make the absorption process more effective in the hydrogel sample.

### Vinasses characterization

The Egyptian Vinasse presented a pH of 4.76, which was higher than that reported by Parsaee et.al [37]. The low pH is due to the presence of acids in the medium, such as acetic, butyric, lactic, and formic acids. The COD value (36.27%) showed that a lot of organic compounds, including polysaccharides, lignin, hemicellulose, proteins, melanoidin, and wax, were still present after distillation in all of the processes [38]. The concentration of potassium is 9.2%, phosphorus is 0.26% and the concentration of nitrogen is 2.88%. Similar data were reported by Hernández et al. [39] in Vinasse extracted from sugarcane.

### Capsulation mechanism of vinasse by CMC/PAA

**Fig. 7:**
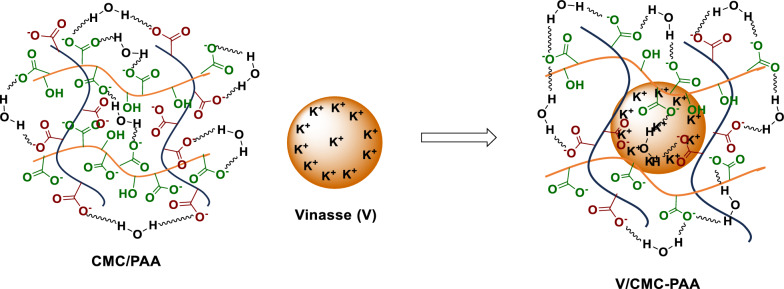
Capsulation of vinasse by CMC/PAA

Vinasses are the liquid waste that results from the end of the distillation process of ethanol [40]. Vinasses contain high concentrations of organic matter, low pH, electric conductivity, and mineral salts [37]. Also, it contains a high concentration of NPK; therefore, it is considered an effective fertilizer in agriculture fields, but it has some problems, such as its high solubility in water, which makes the nutrient compounds lose their value during the leaching process. To overcome this problem, Vinasse was coated with hydrogel, as shown in (Fig. 7). The coating process depends on many forces, such as the hydrogen bonding between the active groups of hydrogel and the active groups present in Vinasse, Hydrophilic-hydrophilic interaction, in addition to the coordination bonds between the elements and the carboxylic groups in the prepared hydrogel, according to this process, the final product acts as a slow-release organic fertilizer.

### Potassium release rate

**Fig. 8:**
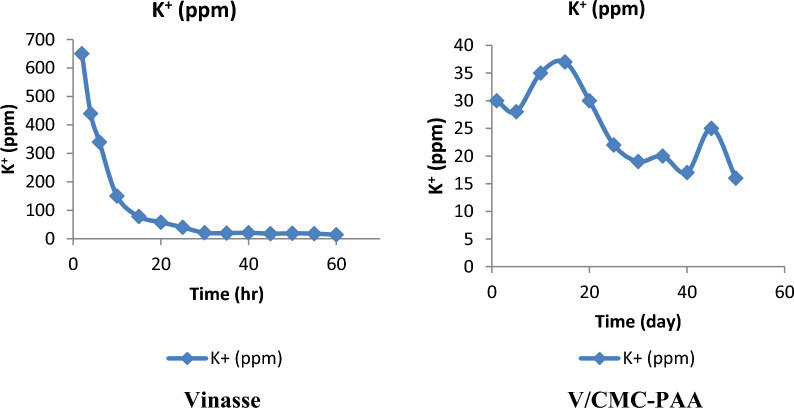
The release rate of potassium K^+^ in leached solution

The results show that the prepared slow release organic fertilizer hydrogel (V/CMC-PAA) is more effective than Vinasse itself. Since, (V/CMC-PAA) can control the release rate of potassium, Table 4A, B investigates the following process for the concentration of potassium in leached water in the cases of using Vinasse alone (4A) and Vinasse with hydrogel (4B). The results illustrate that the concentration of potassium was very high at about 650 ppm after two hours and reached 14 ppm through 60 h, as shown in Table 4A and (Fig. 8). But in the case of using Vinasse with hydrogel CMC/PAA the concentration of potassium in leached water has the same value after 50 days, as shown in Table 4B and (Fig. 8).

**Table 4:** Comparison between the release rate of K^+^ in leached soln. in case of using Vinasse only (A) and using of (V/CMC-PAA) (B)

(A) Potassium content (Vinasse)		(B) Potassium content (V/CMC-PAA)	
Time (h)	K^+^ (ppm)	Time (day)	K^+^ (ppm)
2	650	1	30
4	440	5	28
6	340	10	35
10	150	15	37
15	78	20	30
20	58	25	22
25	40	30	19
30	21	35	20
35	20	40	17
40	21	45	15
45	18	50	14
50	19		
55	18		
60	14

### Evaluation of EC

**Fig. 9:**
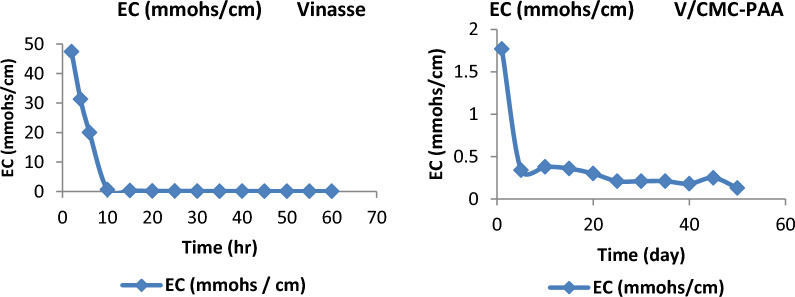
The EC in leached water in case of Vinasse itself and V/CMC-PAA

**Fig. 10:**
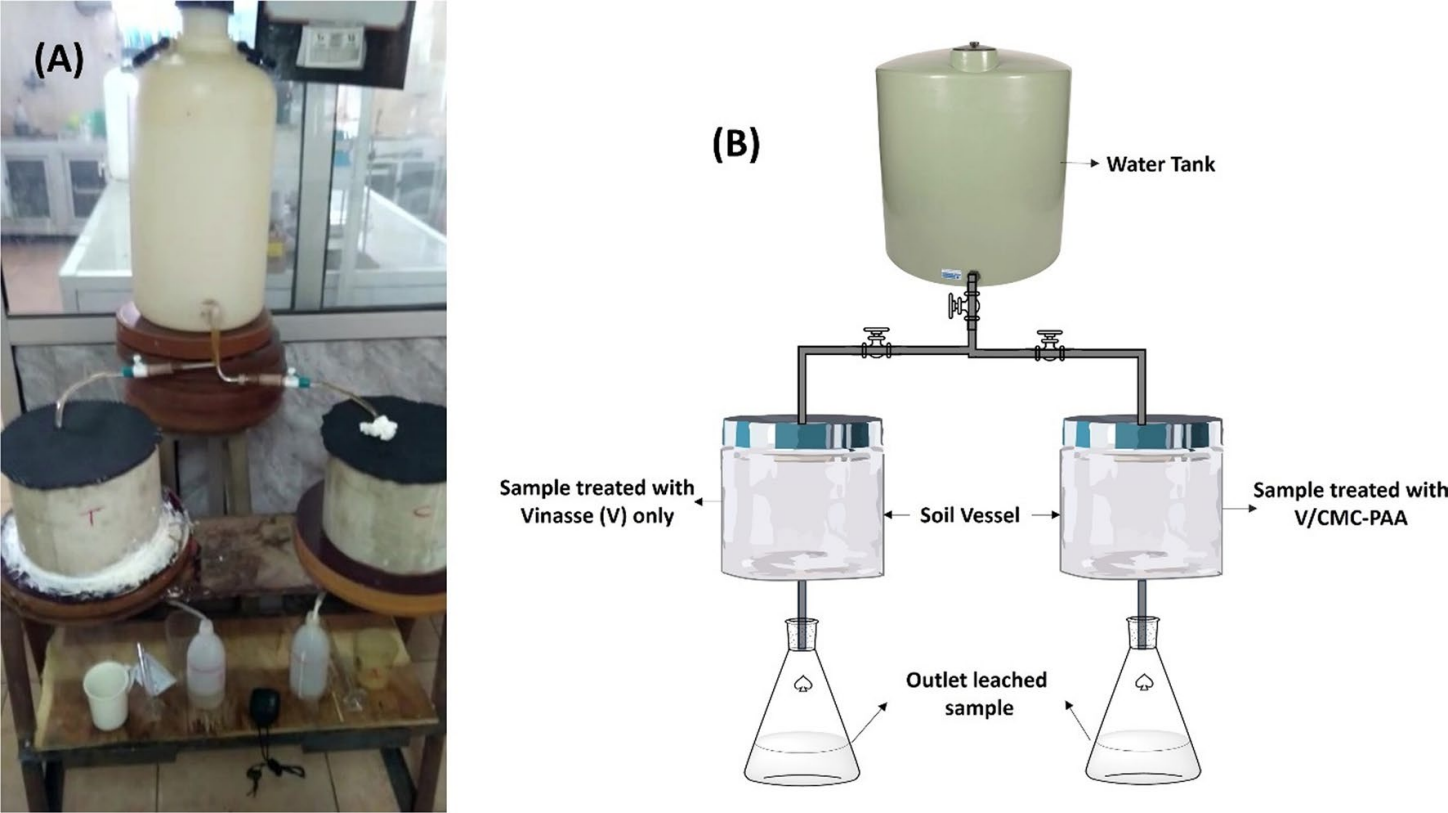
Experimental device setup for Vinasse release study **(A)** Original device, **(B)** Graphic device

Table 5A, B, (Fig. 9) show the EC values for the leached water in the case of using Vinasse only and after applying V/CMC-PAA. The results indicate that the values of EC in the case of using Vinasse itself decreased from 47.4 to 20 mmohs/cm through the first six hours. It's clearly evident that these values show the high EC of Vinasse, which causes the damage of plants by salinity chalk. In contrast, the EC value after applying V/CMC-PAA through the first six hours was 1.77 to 0.35 mmohs/cm. These results showed that by using the hydrogel with Vinasse as SRFH, the plant was protected from the salinity chalk. Therefore, V/CMC-PAA is considered a potential slow release organic fertilizer and meets the requirements of alkaline soil. Moreover, it will be most suitable for Egyptian agricultural fertilizers. All these results were carried out by using the device as shown in (Fig. 10A, B).

**Table 5:** Comparison between the EC in leached water in case of Vinasse itself (A) and V/CMC-PAA (B)

(A) EC (Vinasse)		(B) EC (V/CMC-PAA)	
Time (h)	EC (mmohs/cm)	Time (h)	EC (mmohs/cm)
2	47.4	2	1.77
4	31.3	4	0.34
6	20	6	0.35
10	0.65	10	0.37
15	0.34	15	0.38
20	0.2	20	0.3
25	0.19	25	0.21
30	0.15	30	0.21
35	0.12	35	0.21
40	0.13	40	0.18
45	0.11	45	0.25
50	0.11	50	0.13
55	0.11		
60	0.09

#### The application of (V/CMC-*PAA*) on cane sugar crops

The production of the cane sugar crop was evaluated by examining cane stalk weight, cane stalk height, and the quality of the juice produced through measuring purity and reducing sugar after 360 days. This study was applied to one Hectare from cane sugar crops. This area was divided into two parts. The first part was treated by the prepared V/CMC-PAA, and the second part was treated by Vinasse itself (control). The results show that the characterization of the cane sugar crop treated with V/CMC-PAA as a slow-release organic fertilizer has distinctive properties compared to the crop treated with Vinasse only. The average cane stalk weight in the case of Vinasse itself is 1.28 kg, while in the case of using V/CMC-PAA is 1.74 kg. Also, the average cane stalk height when using Vinasse alone is 2.3 m, while when using coated Vinasse was 2.9 m. The cane juice purity in using Vinasse itself is 84.2 and became 85.6 by using V/CMC-PAA. Moreover, the concentration of reducing sugar in juice in the case of using V/CMC-PAA was 5.16 and in the control was 3.61. The P_2_O_5_ percentage was 160 ppm in the control, while it was 180 ppm in the case of coated Vinasse. The productivity per feddan increased from 32 tons in the case of Vinasse itself to 38 tons after using V/CMC-PAA. This means productivity will increase by about 15% per feddan. In addition, the number of irrigations decreased from 5 to 4 by using V/CMC-PAA Table 6.

**Table 6:** The effect of CMC-PAA hydrogel on sugar cane and cane juice quality after season of cultivation

Analysis	Control (Vinasse)	Treated V/CMC-PAA
Average cane stalk weight—kg	1.28	1.74
Average cane stalk height—m	2.3	2.9
Theoretical sugar yield—%	11.47	12.11
Cane juice purity	84.2	85.6
Reducing sugar % Bx in juice	3.61	5.16
P_2_O_5_ “ppm” in juice	160	180
Number of irrigations (times)	5	4
Productivity per feddan (tons)	32	38

#### The suggested biodegradability of combined SROFH-V/CMC-PAA

Biodegradability is known as a chemical change in compounds due to chemical breakdown processes by microorganisms or enzymes [41]. The biodegradability procedure is based on the category of polymers, such as natural, synthetic, and combination polymers. Combination polymers have been widely investigated for the development of hydrogels with improved properties. There are several methods to measure the biodegradability of hydrogels in soil. Such as the examination of the quantity of evaporated carbon dioxide from the soil and the hydrogel, the determination of the biochemical oxygen demand (BOD) of the improved soil compared to the natural soil, calculating weight loss through the soil burial method, investigating the growth of microbial biomass, determining the changes in the physical properties of the hydrogel with time [42], and using the hydrogel with radioactive ^14^C and tracking the ^14^C in the CO_2_ or CH_4_ released from the metabolism of the polymer. Soil burial testing is the most widely used to study the biodegradability of hydrogels [43]. The degradation rate of a hydrogel was found to be 5.9% under aerobic conditions over about 500 days, as reported by Huettermann et al. [43]. The rates of decomposition of crosslinked polyacrylate hydrogels were reported to be in the range of 1–9% per year. The biodegradation of polyacrylate-based hydrogels occurred by microorganisms that utilized the carbon backbone as a carbon source [44]. Using CMC as a hydrogel incorporating polyacrylic acid increases the biodegradation rate. Secondly, increasing the nutrient amount increased the biodegradation of the hydrogel, as the microbes responsible for the biodegradation process. The bio-based hydrogel CMC/PAA was degradable by using the soil burial method and measuring weight loss [45]. The biodegradation rate was found to be 43.6% after 100 days, and this rate was dependent on the type of soil.

### Economic study

The following economic analysis can be deduced from our previous laboratory-scale experiments: this study was conducted on May 15, 2024.

- The daily production rate of solid Vinasse at the Abo Korkas sugar and distillation factory is 50 tons.
- 2 kg of hydrogel (CMC/PAA) is required to encapsulate 60 kg of Vinasse.
- For 1.0 ton of coated vinasse, the quantity of hydrogel (V/CMC-PAA) is 33.3 kg (2 × 1000/60).
- To manufacture 50 tons of coated Vinasse, an estimated 1670 kg (33.3 × 50) of hydrogel (CMC/PAA) is needed.
- One kilogram of hydrogel costs 0.5 USD.
- Vinasse is priced at $20 per ton.
- About 120 kg of coated Vinasse are added per hectare.
- 4 kg of hydrogel were utilized to encapsulate 120 kg of Vinasse.
- Therefore, the increase in fertilization expenses per hectare will be approximately 2 USD (4 * 0.5).
- Sugar cane harvest costs 45 USD per ton.
- The profit realized from an increase in sugar cane crop productivity is 270 USD per hectare (38−32) × 45.
- The net gain is calculated as the increase in productivity—the increase in fertilization costs (270−2 = 268 USD per hectare).

## Conclusion

In this study, a promising combined hydrogel containing carboxymethyl cellulose and polyacrylic CMC/PAA was prepared and characterized by different methods to be used as a slow-release organic fertilizer hydrogel for Vinasse. The FTIR and XRD analyses proved the formation of hydrogel via the changes that occur in the FTIR spectrum and the XRD pattern. Also, the SEM analysis showed a difference in the morphology structure of Vinasse before and after coating with hydrogel. The Vinasse powder was coated with the prepared hydrogel to control the releasing process of potassium from Vinasse, this technique is known as slow release potassium from Vinasse. Also improving the physical properties of Vinasse such as salinity and porosity. The application field on cane sugar crops proves that the EC value after applying V/CMC-PAA through the first six hours was 1.77 to 0.35 mmohs/cm. This indicates the plant will be saved from the salinity chalk, which is raised by the highly increased EC of Vinasse itself. Therefore, V/CMC/PAA is considered a potential slow release organic fertilizer and meets the requirements of alkaline soil. Also, using V/CMC-PAA can control and keep the potassium as fertilizer for 50 days. Furthermore, the cane stalk weight, cane stalk height, the quality of the juice produced, and the cane juice purity for cane sugar crops were increased. The productivity per feddan was enhanced from 32 to 38 tons after using V/CMC-PAA by about 15% per feddan, and the number of irrigations decreased from 5 to 4 times.

## Data Availability

The datasets used and/or analysed during the current study are available from the corresponding author on reasonable request.
